# Determination of Trace Platinum in Water Samples by Ionic Liquid-Dispersive Liquid–Liquid Microextraction Combined with Graphite Furnace Atomic Absorption Spectrometry

**DOI:** 10.3390/molecules31122020

**Published:** 2026-06-09

**Authors:** Yaqi Liu, Yanyan Huo, Quan Han, Xiaohui Yang

**Affiliations:** School of Chemical Engineering, Xi’an University, Xi’an 710065, China; xaliuyq@163.com (Y.L.); xahquan@163.com (Q.H.); sheepyangxh@163.com (X.Y.)

**Keywords:** ionic liquid, dispersive liquid–liquid microextraction, graphite furnace atomic absorption spectroscopy, response surface optimization, AGREEprep, platinum

## Abstract

A new method has been established for determining trace amounts of platinum in water using ion liquid (IL)-dispersive liquid–liquid microextraction (DLLME) combined with graphite furnace atomic absorption spectroscopy (GFAAS). The method is based on the use of a self-prepared reagent, 5-(5-cyano-2-pyridineazo)-2,4-diaminotoluene (5-CN-PADAT), as a chelating agent, which reacts with Pt(IV) to form a hydrophobic chelate. The extraction solvent is 1-octyl-3-methylimidazolium hexafluorophosphate ([C_8_mim][PF_6_]), and ethyl acetate is used as the dispersive solvent. After the extraction is completed, the extraction phase formed by [C_8_mim][PF_6_] and ethyl acetate has a relatively low viscosity and can be directly used for the determination of GFAAS. A single-factor rotational method was employed to optimize conditions affecting DLLME extraction efficiency. The interactions among the factors affecting DLLME were analyzed using response surface optimization (RSM). Under optimal conditions, platinum concentrations exhibited good linearity within the range of 40–280 ng/mL, with a detection limit of 0.3 ng/mL. AGREEprep was used to discuss the ecological friendliness of the method, demonstrating its low cost, ease of operation, simple equipment requirements, and environmental friendliness. When applied to determining trace amounts of platinum in water samples, the results were satisfactory.

## 1. Introduction

Platinum (Pt) is widely used in industries such as petroleum refining, drug synthesis, and medical device manufacturing [1,2]. Specifically, within the automotive industry, platinum’s exceptional catalytic activity and resistance to sulfur compounds render it the paramount selection for catalysts in automotive exhaust-treatment systems. Three-way catalytic converters utilize platinum group metals (Pd, Rh, and Pt) with catalytic activity as catalysts to reduce harmful gases (such as carbon monoxide and nitrogen oxides) produced by the incomplete combustion of fuel in internal combustion engines, converting them into less harmful substances before emission. Based on this fact, the highest levels of platinum pollution are believed to occur near transportation routes. Although the use of exhaust-treatment systems has reduced greenhouse gas emissions, the catalytic converter surface is exposed to constantly changing chemical, physical, and redox conditions, and the wear and tear on the catalytic converter surface inevitably results in the release of platinum into the environment via exhaust gases. This is the primary anthropogenic source of platinum pollutants entering the environment [3,4,5]. Under the influence of human activities, platinum pollutants are transformed into water-soluble substances and transferred to soil or water bodies, where they can be transmitted through the food chain and accumulate in organisms. Currently, cases of platinum pollutants being detected in organisms have been reported [6,7]. Some studies have also confirmed that platinum metal exhibits cytotoxicity, mutagenicity, and carcinogenicity in organisms [8,9]. Therefore, platinum pollution poses a significant potential threat to organisms, and establishing methods to determine trace amounts of platinum in water is essential.

High-sensitivity detection techniques for platinum include graphite furnace atomic absorption spectroscopy (GFAAS) [10,11], inductively coupled plasma emission spectroscopy (ICP-OES) [12,13], inductively coupled plasma mass spectrometry (ICP-MS) [14,15], X-ray (X-Ray) [16,17], and chemiluminescence (CL) [18,19], etc. Among these, GFAAS stands out for its high sensitivity, minimal sample requirement, and widespread instrument availability. However, platinum concentrations are extremely low in environmental water samples, often below the detection limits of the instrument. Additionally, the complex matrix of water samples can significantly impact the reliability of detection results. A common approach to address these issues is to employ extraction techniques before quantitative analysis of metal elements.

Common platinum extraction techniques include: solid-phase extraction [20,21], liquid-phase extraction [22,23], ion exchange [24,25], and coprecipitation [26,27], among others. In recent years, there has been growing interest in microextraction techniques that require low sample volumes, simple equipment, and minimal reagent consumption [28,29]. Among these, the most developed microextraction techniques are solid-phase microextraction [30] and liquid-phase microextraction [31]. Dispersive liquid–liquid microextraction (DLLME) is a type of liquid-phase microextraction first proposed in 2006 [32]. The principle involves adding a dispersive solvent to the extraction system to disperse the extraction phase into countless tiny droplets, thereby increasing the contact area between the extraction agent and the aqueous phase and improving extraction efficiency. This method has the advantages of simple equipment, convenient operation, minimal reagent consumption, and short processing time. However, an inherent drawback of traditional DLLME is that it mostly uses toxic, highly volatile chlorinated reagents (such as chloroform and chlorobenzene) as the extraction solvent, which can easily cause harm to human health through airborne transmission. Ionic liquids, with a vapor pressure close to 0, perfectly compensate for this deficiency.

Ionic liquids (ILs) are liquids composed of cations and anions at room temperature, characterized by low volatility, high thermal stability, good conductivity, and strong structural design capability. They are widely used in the extraction of precious metals in separation and enrichment applications [33]. However, the high viscosity and density of most ILs limit their application in separation fields. For example, in the extraction field, the cations in imidazole-based ILs tend to adsorb onto devices with electrostatic adsorption properties, resulting in reduced IL mass reaching the analyzer and weakened analytical signals [34]. Additionally, the high viscosity of ILs causes them to adsorb onto plastic/glass tube walls (such as glassware used in extraction processes and capillary tubes for sample addition), making quantitative transfer difficult. Most solutions involve including additional steps after metal extraction (such as diluting with organic solvents like methanol or acetone or adding a counter-extraction step) before measurement [35]. However, these cumbersome procedures have led to doubts about the green, safe, and convenient characteristics of IL-based extraction systems, prompting some researchers to explore alternatives [36]. Zheng [37] et al. selected a hydrophobic IL with a density lower than water. Ultrasonic assistance was used to create a cavitation effect between the IL and the aqueous phase, increasing the contact area between the two phases while also reducing IL adsorption on the vessel walls to some extent. Finally, through reverse extraction, Pb(II) chelates were transferred from the IL to 0.5 mol/L HNO_3_, and GFAAS technology was used to determine lead (II) levels in human urine and blood, yielding satisfactory results. Similarly, Shoichi Katsuta [38] noted that some ILs exist as solids or highly viscous liquids at room temperature, requiring dilution with organic solvents prior to extraction. An IL with low viscosity and strong hydrophobicity, trioctylammonium nitrate ([HTOA][NO_3_]), was prepared experimentally. It demonstrated extremely high extraction capacity for Pd(II) and Pt(IV) in dilute HCl solution without dilution.

Due to the harmful effects of traditional DLLME extraction solvents on human health and the environment, ILs have been widely adopted as alternative extraction agents in the field of extraction. The organic phase obtained after DLLME can meet the sample injection requirements of GFAAS. Additionally, the reduced use of chemical reagents decreases the cost and environmental impact of sample preparation, aligning with the principles of green sample pretreatment. Therefore, the combination of IL-DLLME and GFAAS is highly compatible.

**Figure 1:**
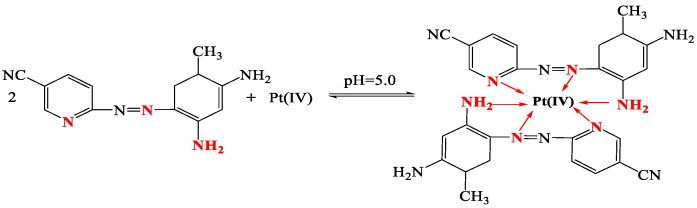
Chemical structure of 5-CN-PADAT and its Pt(IV) chelate.

This study aims to establish a new method for the determination of trace amounts of platinum in water using IL-DLLME, which is simple, requires minimal reagent consumption, and is environmentally friendly. A self-prepared reagent, 5-(5-cyano-2-pyridine azo)-2,4-diaminotoluene (5-CN-PADAT), is used as the chelating agent. This chelating agent exhibits high selectivity in metal-ion recognition and extraction due to its unique coordination structure, and has been successfully applied in the quantitative analysis of various trace metals [39]. This class of reagents has a unique “N, N, N” coordination structure, forming a stable hydrophobic chelate with platinum in a 2:1 ratio. The reaction process is shown in Figure 1 [40], and after extraction and separation, the metal chelate can be transferred from the aqueous phase to the extractant phase. The experiment used ILs as the extraction solvent, DLLME as the extraction method, and GFAAS as the detection method to determine trace platinum in environmental samples. A single-factor rotation method was used to optimize the factors affecting liquid–liquid microextraction. In the response surface optimization method, the interaction of two factors in DLLME was represented by a three-dimensional graph, and the feasibility of the experimental design was verified by analysis of variance (ANOVA). This method was compared with previously reported DLLME extraction methods for platinum, and its ecological usability was assessed using the AGREEprep metric [41]. Finally, this method was applied to the determination of water-soluble trace Pt(IV) species in environmental water samples.

## 2. Results

### 2.1. Single-Factor Rotation Method

#### 2.1.1. Selection of Extraction-Solvent Types and Volume

**Figure 2:**
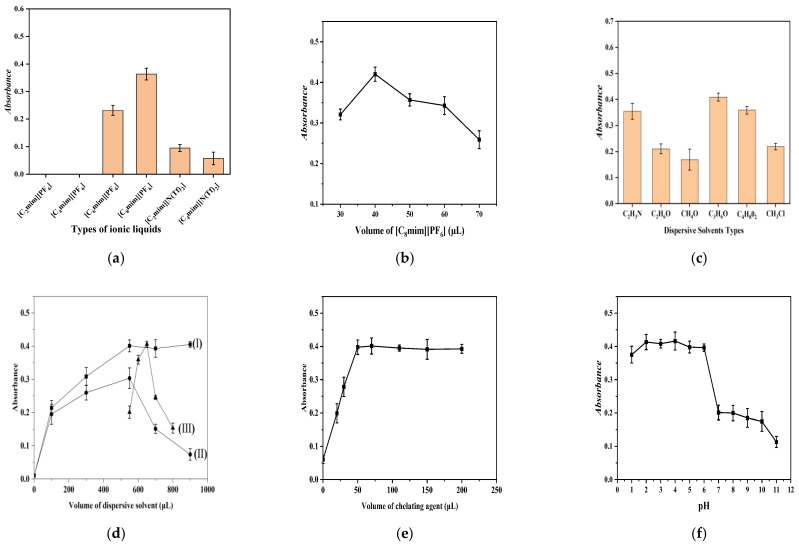
The influence of various factors on absorbance: (**a**) types of ionic liquids; (**b**) volume of ionic liquid; (**c**) types of dispersive solvents; (**d**) volume of dispersive solvent; (**e**) volume of chelating agent; (**f**) pH.

The appropriate type of extraction agent ensures better separation of metal complexes. Ionic liquids were used as the extraction solvent in this experiment, with their extraction mechanism following the ion-exchange mechanism [42,43]. Since platinum complexes carry a positive charge under acidic conditions, they can exchange with cations in the ionic liquid, achieving phase transfer of metallic platinum. Six commonly available imidazole-based ionic liquids widely used in extraction applications were selected. The relationship between different extraction-solvent types and the absorbance (A) of the GFAAS measurement signal was investigated, with results shown in Figure 2a. The results indicate that the extraction solvents [C_2_mim][PF_6_] and [C_4_mim][PF_6_] did not exhibit phase separation after centrifugation, while the other four ILs showed phase separation after extraction. The reasons are that (i) the hydrophobicity of ILs decreases as the carbon-chain length of the cation shortens. Therefore, the shorter-chain [C_2_mim][PF_6_] and [C_4_mim][PF_6_] remain dissolved in the aqueous phase after extraction [44]. (ii) The hydrophobicity of ILs increases with the size of the anion. Since the ionic radius of [N(Tf)_2_]^−^ is larger than that of [PF_6_]^−^, even with shorter carbon chains in the cation, it exhibits better hydrophobicity [35]. As shown in Figure 2a, [C_8_mim][PF_6_] exhibits the best extraction efficiency for platinum complexes.

Sufficient amounts of extraction solvent ensure that metal chelates are completely transferred to the extraction phase. The experiment investigated the effect of IL volume between 30 and 70 μL on absorbance (experiments were not conducted below 30 μL due to the small recovery volume of the organic phase and difficulty in recovery). The results are shown in Figure 2b. The results indicate that when the IL volume reaches 40 μL, the absorbance reaches its maximum, indicating that the platinum chelates are completely extracted into the extraction phase. After extraction, due to the small recovery volume of the extraction phase, subsequent experimental operations became difficult. Therefore, the volume of [C_8_mim][PF_6_] selected for the experiment was 50 μL.

#### 2.1.2. Selection of Dispersive-Solvent Types and Volume

Different types of dispersive solvents affect the dispersion degree of the extraction solvent in the aqueous phase, directly impacting extraction efficiency. The selection criteria for DLLME dispersive solvents are as follows: the dispersive solvent must be polar and miscible with the extraction solvent, soluble in water, and incapable of reacting chemically with the analyte. ILs possess high polarity and are miscible with most organic reagents. During the extraction process, the addition of the dispersant causes the extraction solvent IL to disperse into countless tiny droplets in the aqueous phase, increasing the contact area with the aqueous phase and thereby accelerating the phase transfer rate of metal complexes. The experiment investigated the changes in absorbance when six organic reagents were used as the dispersive solvent, with the results shown in Figure 2c. The results show that the absorbance decreases in the following order when different dispersants are used: acetone (C_3_H_6_O) > acetonitrile (C_2_H_3_N) = ethyl acetate (C_4_H_8_O_2_) > dichloromethane (CH_3_Cl) > ethanol (C_2_H_6_O) > methanol (CH_4_O). After extraction, 50 μL of acetonitrile was used as a diluent to reduce the viscosity of the IL. It was found that when ethyl acetate was used as the dispersive solvent, the recovery volume of the extraction phase was 120 μL. When the other five reagents were used as dispersing solvents, the IL recovery volume was 100 μL. This is because ethyl acetate is poorly soluble in water and forms the extraction phase together with the IL after extraction. Considering that an increase in the extraction-phase volume would reduce the platinum concentration and cause experimental errors, the amounts of acetone and ethyl acetate were optimized in subsequent experiments.

Sufficient amounts of dispersive solvent ensure complete dispersion of the extraction solvent. The experiment investigated the effect of dispersive solvents (acetone and ethyl acetate) on the absorbance of the instrument signal value. The results showed that: Figure 2d-(I) when the amount of acetone was between 600 and 900 μL, the absorbance reached a maximum and remained constant, with a recovered extract-phase volume of 100 μL. Figure 2d-(II) shows that the absorbance reached a maximum at an ethyl acetate volume of 550 μL, with a recovered extraction-phase volume of 120 μL. At this point, the recovery volume is greater than 100 μL, indicating that the organic phase is a mixture of ethyl acetate and ionic liquid. To simplify the experimental steps, the changes in absorbance were investigated for ethyl acetate volumes between 550 μL and 800 μL without adding a diluent; these limits were selected because when the ethyl acetate volume was less than 550 μL, the IL viscosity was too high and adhered to the test tube wall, making quantitative transfer impossible, and when the volume exceeded 800 μL, the extract phase floated on the upper layer of the aqueous phase, resulting in lower measured absorbance, and no further experiments were conducted. The results are shown in Figure 2d-(III). The results indicate that when the ethyl acetate volume was 650 μL, the absorbance reached its maximum, similar to when acetone was used as the dispersive solvent, and the extract-phase recovery volume was 100 μL. The experiment selected 650 μL of ethyl acetate as the dispersive solvent.

#### 2.1.3. Volume of Chelating Agent

A sufficient volume of chelating agent ensures that platinum in the sample is completely complexed. The reagent 5-(5-cyano-2-pyridineazo)-2,4-diaminotoluene (5-CN-PADAT) was selected as the chelating agent in this experiment. This chelating agent is dissolved in ethanol. If the concentration of this reagent is too high, the amount of ethanol introduced into the system will be excessive, affecting the formation of micelles and the extraction phase. If the chelating agent concentration is too low, 5-CN-PADAT cannot be completely dissolved. Considering all factors, the chelating agent concentration selected for the experiment was 5 × 10^−4^ mol/L. The experiment investigated the changes in absorbance with chelating agent volume ranging from 0 to 200 μL, with results shown in Figure 2e. The results indicate that the absorbance reaches a maximum when the chelating agent volume is between 50 and 200 μL (no experiments were conducted beyond 200 μL). At this point, the platinum in the sample has completely formed hydrophobic complexes. The chelating agent volume used in the experiment was 70 μL.

#### 2.1.4. Effect of pH

The acidity of the solution affects the formation and hydrophobicity of platinum complexes, thereby influencing extraction efficiency. The chelating agent 5-CN-PADAT contains three nitrogen atoms that can bind with protons, and platinum complexes also have unpaired nitrogen atoms. Therefore, the acidity of the solution affects the extraction efficiency. The experiment investigated changes in absorbance within a pH range of 1–11, with results shown in Figure 2f. The results indicate that when pH is between 2 and 6, absorbance reaches its maximum, at which point the chelating agent exhibits the strongest complexation ability with Pt. The experiment selected an HAc-NaAc buffer solution with pH = 5.0.

#### 2.1.5. Influence of Other Factors

Heating Time: An appropriate boiling water-bath time ensures that the chelate is completely chelated with Pt. Since platinum is an inert metal, external heating is often required to facilitate the reaction between the metal and 5-CN-PADAT in the complexation reaction. The experiment investigated the changes in the instrument signal value absorbance when the boiling water-bath time ranged from 0 to 10 min. The results showed that when the heating time was between 2 and 10 min, the absorbance reached its maximum, indicating that Pt and the chelating agent could be completely chelated. The experiment selected a heating time of 4 min.

Extraction Time: Sufficient extraction time ensures that the chelate is completely transferred to the precipitate phase. The experiment investigated changes in absorbance over extraction times ranging from 0 to 5 min. Results showed that absorbance reached a maximum and stabilized between 3 and 5 min, indicating that the platinum chelate was completely separated into the precipitate phase. The experiment selected an extraction time of 4 min.

Centrifugation Time: Sufficient centrifugation time ensures that the organic phase completely aggregates at the bottom of the centrifuge tube, facilitating separation. The experiment investigated changes in absorbance over 0–5 min of centrifugation at a centrifuge speed of 4000 r/min. The results showed that absorbance reached a maximum and remained essentially constant when the centrifugation time was between 2 and 5 min. The experiment selected a centrifugation time of 3 min.

#### 2.1.6. Study of Interfering Ions

Under optimal conditions, the influence of various interfering species on the determination of 650 ng of platinum was investigated, and a relative error of ±5% in the absorbance was considered tolerable. The results of the tolerance limits of various foreign species were: 5000 times: K^+^, Mg^2+^, Ca^2+^, Al^3+^, and I^−^; 1000 times: NH_4_^+^, Mn^2+^, Zr(IV), La^3+^, Sr^2+^, PO_4_^3−^, Ba^2+^, Ni^+^, F^−^, SO_4_^2−^, Zn^2+^, and Sb^3+^; 200 times: Cr(VI), Hg^2+^, Co^2+^; 100 times: Bi^3+^, Cd^3+^, and As^3+^; 20 times: Pd^2+^, Au^+^, and Ir^3+^; 5 times: Fe^3+^, Cu^2+^, Ag^+^, Rh(III), and Os(IV).

### 2.2. Response Surface Optimization Method

Response surface optimization (RSM) is a statistical method [45,46]. Its principle is to construct a multivariate and continuous mathematical model through a limited number of experiments, and this model is usually a polynomial equation. The establishment of this method is more conducive to understanding the relationship among various variables in the experiment and finding the optimal experimental conditions. This work aims to fit the complete quadratic-equation model, obtain the optimal experimental conditions, verify the optimal conditions obtained by the single-factor rotation method, and analyze the interaction relationship between various variables in the IL-DLLME and the instrument signal value A. This experiment was conducted using Design Expert 13 software. In order to reduce the number of factors in the experimental design, the initial conditions were set as follows: [C_8_mim][PF_6_] as the extraction solvent; ethyl acetate as the dispersive solvent; boiling water-bath time is 4 min; the mixture is evenly mixed on the vortex mixer for 3 min; and the centrifugation time after extraction is 3 min. Regarding the four factors affecting the extraction efficiency of DLLME: IL volume (30–70 μL), ethyl acetate volume (500–800 μL), pH (2–10), and chelating agent volume (0–160 μL). A total of 29 groups of experiments were determined in the Box–Behnken Design works. Under the given conditions, extraction was carried out on 5 mL of a 150 ng/mL platinum aqueous solution. The signal value A was determined by GFAAS. All experiments were conducted three times.

The validity of model fitting was evaluated using Analysis of Variance (ANOVA) and the coefficient of determination (R^2^). As seen in Table 1, the F-value of the model obtained is 101.78, and the *p*-value is less than 0.0001, indicating that the fitted model is significant. The correlation coefficient R^2^ is 0.9903 and the adjusted R^2^ is 0.9805, which proves that the experimental results are basically consistent with the theoretical results, indicating that the model can reflect and predict the influence of condition changes on the signal values analyzed by the instrument. The multiple quadratic regression Equation (1) of the signal value absorbance and various factors affecting DLLME can be obtained by using software. In the quadratic equation, variables with positive coefficients have an enhancing effect on the signal value A, while variables with negative coefficients have a reducing effect on the signal value A. It can be determined from the equation that:

1. The volume of the extraction solvent (F_1_), the volume of the dispersive solvent (F_2_), and pH (F_3_) have negative effects, while the volume of the chelating agent (F_4_) has a positive coefficient;
2. The secondary variables F_1_, F_2_, F_3_, and F_4_ in this model significantly affect the change in absorbance;
3. The main interacting variables that affect the variation in the maximum signal value A in modeling are F_2_ and F_4_.
  A = 0.43 − 0.031 F_1_ − 0.038 F_2_ − 0.086 F_3_ + 0.11 F_4_ + 0.086 F_1_F_2_ + 0.017 F_1_F_3_ − 0.012 F_1_F_4_ − 0.025 F_2_F_3_ − 0.086 F_2_F_4_ − 0.065 F_3_F_4_ − 0.068 F_1_^2^ − 0.059 F_2_^2^ − 0.094 F_3_^2^ − 0.20 F_4_^2^ (1)

**Figure 3:**
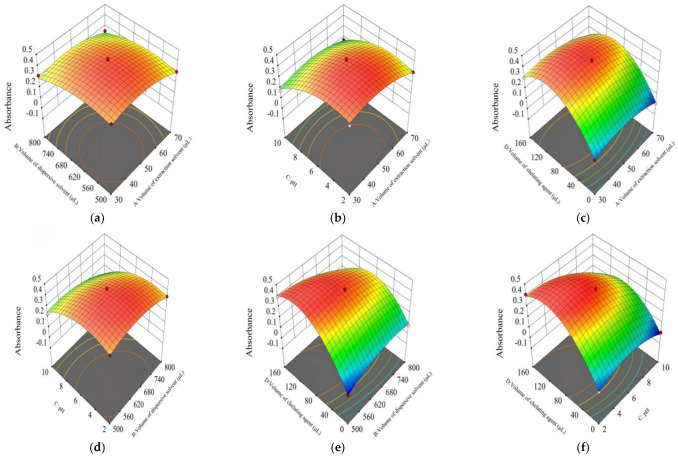
Response surface plots showing the interrelationships between: (**a**) extraction solvent volume and dispersive solvent volume; (**b**) extraction solvent volume and buffer solution pH; (**c**) extraction solvent volume and chelating agent volume; (**d**) dispersive solvent volume and buffer solution pH; (**e**) dispersive solvent volume and chelating agent volume; (**f**) buffer solution pH and chelating agent volume.

The response surface optimization method was adopted to optimize the key factors, and the modality of the response surface was explained. The interaction of the four factors is presented in the three-dimensional Figure 3. Figure 3a analyzes the influence of the amount of extraction solvent and dispersive solvent on the instrument signal value A. The high signal value A tends to have a lower amount of extraction solvent and dispersive solvent, as a smaller volume of the metal chelate concentration in the extraction phase is more fully dispersed, resulting in a higher signal value obtained. Figure 3b analyzes the influence of the amount of extraction solvent and pH on absorbance A. When the pH is greater than 7, the metal ions undergo hydrolysis, resulting in a decrease in the extraction effect and signal value. Figure 3c analyzes the influence of the volumes of the extraction solvent and chelating agent on A. Within the experimental range, a smaller volume of the extraction solvent and a larger volume of the chelating agent can yield larger signal values. When the amount of chelating agent is small, it cannot completely complex with metal ions, resulting in incomplete extraction and a decrease in absorbance. Figure 3d analyzes the interaction between the amount of dispersive solvent and pH on A. Within the experimental range, a smaller pH and the amount of dispersing solvent can obtain the highest signal value A. Figure 3e analyzes the interaction between the volume of the dispersive solvent and the volume of the chelating agent on A. Within the experimental range, the highest instrument signal value can be obtained by reducing the volume of the dispersive solvent and increasing the volume of the chelating agent. The interaction between the pH of the buffer solution and the volume of chelating agent on A was analyzed, as shown in Figure 3f. Within the experimental range, higher signal values tend to have a smaller pH and a higher volume of chelating agent. The optimal experimental conditions predicted by the model are as follows: the volume of the extraction solvent is 50 μL, the volume of the dispersive solvent is 653 μL, pH = 2.8, and the volume of the chelating agent is 128 μL.

### 2.3. Method Validation

The optimal conditions obtained using response surface optimization and the single-factor rotation method were applied to parallel measurements of a platinum standard solution with an absolute concentration of 750 ng, each performed three times, with the average value taken. The relative standard deviation was 4.6%, indicating that the results obtained using this method were comparable to those obtained using the single-factor rotation method. Under the experimental conditions optimized using the single-factor rotation method, the relationship between platinum mass concentration and analytical signal was investigated. The method demonstrated good linearity over 40–280 ng/mL (y = 0.00334x − 0.0302, R = 0.9929). Residuals were randomly distributed between −0.0723 and +0.0401, indicating no significant systematic error. The 95% confidence intervals for the slope and intercept were [0.00273, 0.00395] and [−0.1312, 0.0708], respectively. The narrow slope interval further validates the high precision and stability of the method. Seven sets of blank samples were analyzed in parallel, with a method detection limit of 0.3 ng/mL, calculated using the detection limit formula 3σ/k (where σ is the standard deviation of the blank sample’s absorbance, and k is the slope of the calibration curve). Seven determinations were performed on a platinum standard solution of 150 ng/mL, with a relative standard deviation (RSD) of 4.1%. For platinum concentrations ranging from 0 to 2000 ng/mL, the standard curve for platinum without extraction was determined experimentally, with the linear regression equation being A = 0.00008c − 0.0018 and a correlation coefficient of 0.9996. The enrichment factor (EF) was calculated by dividing the slope of the working curve after enrichment by the slope of the standard curve before enrichment, resulting in an EF of 42 for this method.

Table 2 summarizes the advantages of the proposed method: (i) limit of detection (LOD); (ii) required sample volume; (iii) enrichment factor (EF); (iv) consumption index (CI) [47]—this parameter is defined as the ratio of sample volume (μL) to EF and can be used to evaluate the extraction performance of the method; (v) AGREEprep evaluation, the green metric value of the proposed method, calculated using the AGREEprep software proposed by Wojciech Wojnowski. The environmental friendliness of sample preparation was assessed according to the requirements of green analytical chemistry. The proposed method was compared with previously reported studies on platinum extraction using DLLME. It can be seen that the extraction efficiency (CI values) of this method is superior to that reported in [48,49]. Although it is lower than that reported in [50], that method uses mobile phases in LC that typically contain organic solvents, resulting in a large amount of waste solvents, which does not align with the development needs of green analytical chemistry. From an ecological perspective, the green metric value of the proposed sample preparation method—calculated based on the ten indicators of AGREEprep—is 0.38, which is higher than those of the other three methods. A higher green metric value indicates that the method possesses better environmental friendliness. Additionally, the proposed method uses ethyl acetate as the dispersing solvent, which enhances IL extraction efficiency while reducing dilution steps, providing a potential solution to the issue of IL adhesion during extraction.

### 2.4. Sample Analysis

Platinum (IV) in surface-water samples (including river and lake water) was determined under the optimal conditions obtained via the single-factor optimization method. To verify the reliability of the method, the accuracy of the method can be preliminarily evaluated based on the recovery rate of platinum from water samples and the results of measurements with standard water samples. The optimal conditions obtained using the single-factor rotation method were applied to determine trace amounts of platinum in water samples (a–d). Spiked experiments were conducted by adding different concentrations of platinum standard solutions to water samples (a–d). The experimental results and calculated spiked recovery rates are shown in Table 3. The results indicate that the spiked recovery rates for the four groups of water samples ranged from 92.8% to 102.3%, demonstrating that the method is feasible for extracting platinum from water and is not interfered with by other matrix components.

## 3. Materials and Methods

### 3.1. Regents

1-Ethyl-3-methylimidazolium hexafluorophosphate ([C_2_mim][PF_6_]), 1-Butyl-3- methylimidazolium hexafluorophosphate ([C_4_mim][PF_6_]), 1-hexyl-3-methylimidazole hexafluorophosphate ([C_6_mim][[PF_6_]), 1-octyl-3-methylimidazole hexafluorophosphate ([C_8_mim][PF_6_]), 1-ethyl-3-methylimidazole bis(trifluoromethanesulfonyl)imide salt ([C_2_mim][N(Tf)_2_]), and 1-butyl-3-methylimidazole bis(trifluoromethanesulfonyl)imide ([C_4_mim][N(Tf)_2_]) were all purchased from Shanghai Aladdin Bio-Chem Technology Co., Ltd., Shanghai, China.

Methanol, ethanol, acetone, acetonitrile, ethyl acetate, dichloromethane, and acetone were purchased from Shanghai Guoyao Group Chemical Co., Ltd., Shanghai, China, and all reagents were of analytical grade.

Platinum Stock Solution: 1000 μg/mL, medium 1.0 mol/L nitric acid, purchased from the National Center for Nonferrous Metals and Electronic Materials Analysis and Testing in Beijing, China, diluted with water to the desired concentration as needed.

Buffer Reagent: Acetic Acid–Sodium Acetate Buffer Solution (pH = 2–6): Prepared by mixing 0.2 mol/L HAc with 0.2 mol/L NaAc, and calibrated using a pH meter. Britton–Robinson Buffer Solution (pH = 7–9): Prepared by mixing a ternary acid solution (phosphoric acid, acetic acid, and boric acid, each at a concentration of 0.04 mol/L) with 0.2 mol/L NaOH, and calibrated using a pH meter.

Ethanol solution of 5-CN-PADAT, concentration 5 × 10^−4^ mol/L. At 273.15 K, this substance was prepared by mixing 5-cyano-2-aminopyridine diazonium salt with 2,4-diaminotoluene. The detailed synthesis method for 5-CN-PADAT is described in reference [51].

### 3.2. Instruments

A pH meter (PHS-3C, Shanghai Sanshin Instrument Co., Ltd., Shanghai, China); vortex mixer (VORTEX GENIUS 3, IKA, Guangzhou, China); centrifuge (TD4Z, Kaida Industrial Development Co., Ltd., Changsha, China); ultra-pure water preparation using a water purifier (Milli-Q IQ 7000, Millipore, Burlington, MA, USA). All glassware used in the experiment was soaked in 5% nitric acid for more than 24 h, followed by three or more rinses with ultra-pure water.

Platinum was determined using a graphite furnace atomic absorption spectrometer (PinAAcle 900T, PerkinElmer, Shelton, CT, USA), which is equipped with longitudinal Zeeman background correction: the graphite tube is heated laterally, significantly reducing matrix effects and providing twice the light intensity compared to other Zeeman systems, thereby achieving the best detection limit. Sample introduction was performed using the graphite furnace automatic sampler (AS900, PerkinElmer, CT, USA). The Tube View feature in the Syngistix for AA operating system allowed the observation of the automatic sampler needle transferring the sample into the graphite furnace, as well as monitoring of the drying and pyrolysis processes during analysis. Argon gas with a purity greater than 99.999% (Xi’an Tenglong Chemical Co., Ltd., Xi’an, China) was used as a protective gas to remove volatile impurities generated by pyrolysis on the surface of the graphite tube. A platinum hollow cathode lamp (Array Research Group Pt., Ltd., Beijing, China) was used as the light source, with a lamp current of 10 mA. The measurement wavelength for Pt is 265.94 nm, and the spectral bandwidth slit is 0.7 nm. The graphite furnace heating program is shown in Table 4.

### 3.3. Experimental Method

Take 5 mL of standard solution in a 10 mL conical centrifuge tube, add 1 mL of pH = 5.0 HAc-NaAc buffer solution and 70 μL of 5-CN-PADAT ethanol solution in sequence, mix well, heat in a boiling water bath for 4 min, then cool to room temperature under running water. Add 50 μL of [C_8_mim][PF_6_] as the extraction solvent and 650 μL of ethyl acetate as the dispersive solvent. Mix thoroughly for 4 min using a vortex mixer. Centrifuge at 4000 r/min (g-force 2325× *g*) for 3 min to achieve phase separation. After removing the upper aqueous phase, transfer 100 μL of the organic phase from the bottom of the centrifuge tube to the autosampler using a micropipette, and determine using GFAAS.

## 4. Conclusions

A new method for the determination of trace platinum in water samples by ion liquid-dispersive liquid–liquid microextraction combined with graphite furnace atomic absorption spectrometry was proposed in this experiment. The self-made reagent 5-CN-PADAT was used as a chelating agent, which could form a hydrophobic chelate with platinum. During the DLLME process, ethyl acetate (dispersive solvent) and [C_8_mim][PF_6_] (extraction solvent) formed an extraction phase together after centrifugation. This operation not only accelerated the atomization degree of the extraction solvent in the aqueous phase and shortened the extraction time, but also overcame the difficulty of the high viscosity of the IL, which was not easily directly transferred or adsorbed on the instrument components, causing the loss of the extraction agent. It also eliminated the dilution operation step and simplified the experimental process. The experimental conditions of IL-DLLME were optimized by the single-factor rotation method, and the interaction of two factors in the DLLME system was explored by the response surface optimization method. The environmental performance of the method was evaluated by AGREEprep. The method was successfully applied to the determination of trace platinum in water samples, and the recovery rate of the standard addition was between 92.8% and 102.3%. This method has the advantages of being environmentally friendly, low cost, time-saving, and convenient to operate.

## Figures and Tables

**Table 1:** ANOVA for response surface quadratic model on extraction of Pt(IV) ion.

Source	Sum of Squares	df	Mean Square	F-Value	*p*-Value
Model	0.6222	14	0.0444	101.78	<0.0001
F_1_	0.0113	1	0.0113	25.88	0.0002
F_2_	0.0174	1	0.0174	39.76	<0.0001
F_3_	0.0879	1	0.0879	201.39	<0.0001
F_4_	0.1574	1	0.1574	360.54	<0.0001
F_1_F_2_	0.0002	1	0.0002	0.4268	0.5242
F_1_F_2_	0.0012	1	0.0012	2.73	0.1205
F_1_F_4_	0.0005	1	0.0005	1.24	0.2836
F_2_F_3_	0.0025	1	0.0025	5.65	0.0323
F_2_F_4_	0.0297	1	0.0297	68.07	<0.0001
F_3_F_4_	0.0170	1	0.0170	38.95	<0.0001
F_1_^2^	0.0296	1	0.0296	67.70	<0.0001
F_2_^2^	0.0229	1	0.0229	52.56	<0.0001
F_3_^2^	0.0569	1	0.0569	130.38	<0.0001
F_4_^2^	0.2735	1	0.2735	626.37	<0.0001
Residual	0.0061	14	0.0004		
Lack of Fit	0.0056	10	0.0006	4.80	0.0719

**Table 2:** Comparison of the proposed method with previous works for platinum determination based on DLLME.

Method ^a^	Sample	Extraction Solvent ^b^	Dispersive Solvent	Chelating Agent ^c^	Sample Volume (mL)	EF ^d^	LOD (ng/mL)	CI (μL)	AGREEprep Evaluation	Ref.
ICP-OES	Water	BmimPF_6_	methanol	BDPPIMPF_6_	4	15	1	270	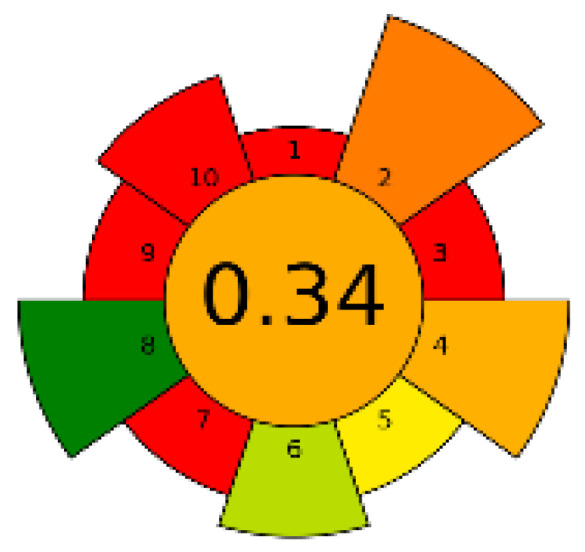	[47]
ICP-QMS	Water and Drug	CHCl_3_	methanol	Aliquat^®^ 336	35	75	4 × 10^−5^	466	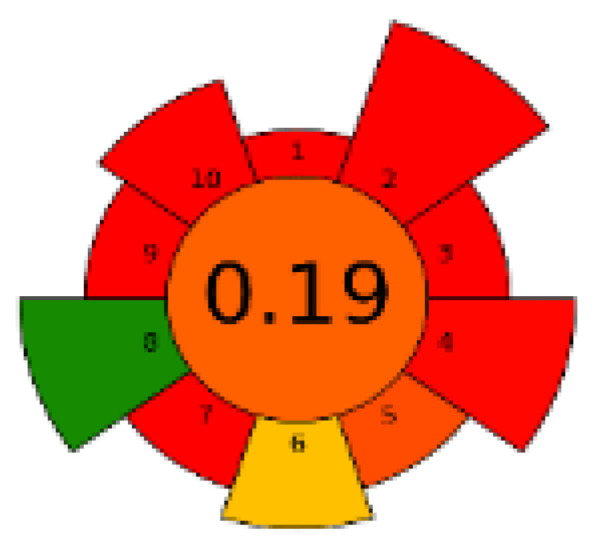	[48]
HPLC	Water	CCl_4_	acetone	PAN	5	119	0.3	42	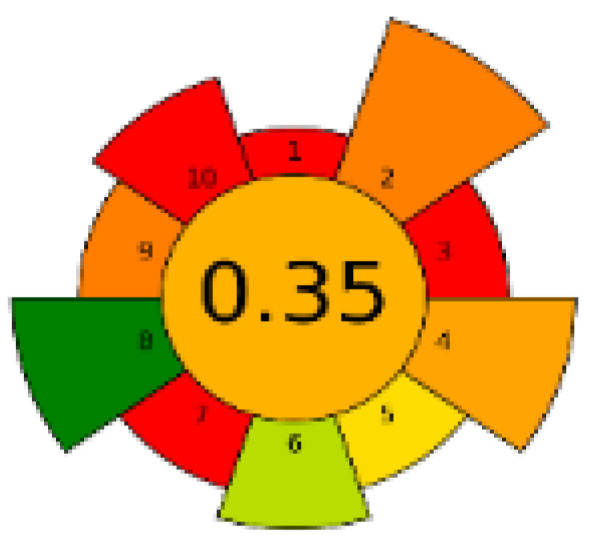	[49]
GFAAS	Water	[C_8_mim] [PF_6_]	ethyl acetate	5-CN-PADAT	5	42	0.3	119	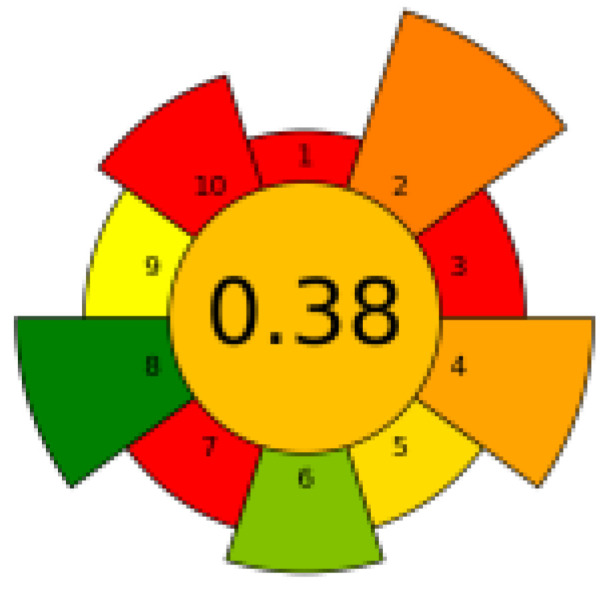	This work

^a^ ICP-QMS: inductively coupled plasma–quadrupole mass spectrometry. HPLC: high-performance liquid chromatography. ^b^ BmimPF6: 1-butyl-3-methylimidazolium hexafluorophosphate. ^c^ PAN: 1-(2-pyridylazo)-2-naphthol. Aliquat^®^ 336: tricaprylmethylammonium chloride. BDPPIMPF6: 1-butyl-2-diphenylphosphino-3-methylimidazolium hexafluorophosphate. ^d^ EF: enrichment factor.

**Table 3:** Determination results of platinum in samples (mean ± 95% confidence level, *n* = 3).

Sample *	Measurement (ng/mL)	Added (ng/mL)	Measurement ** (ng/mL)	Recovery (%)
Standard sample ^a^	82.6 ± 3.1	100	180.5 ± 4.3	100.3
Water ^b^	<DL	200	185.6 ± 5.8	92.8
Water ^c^	<DL	200	204.5 ± 4.4	102.3
Water ^d^	<DL	200	195.4 ± 5.5	97.7

* Sample (^a^) was purchased from Northern Great Metrology Group (Beijing, China). Product number: BWB2236-2016. Water samples (^b–d^) were provided by the Shaanxi Provincial Bureau of Hydrological and Water Resources Survey. Sample information: (^a^) Platinum standard concentration: 80 ± 4 ng/mL. (^b^) Yuxi (Heyang County, Weinan City, China): Cu < 0.05 mg/L, Zn < 0.05 mg/L, Fe < 0.03 mg/L, Mn < 0.01 mg/L, Pb < 0.0025 mg/L, Cd < 0.0005 mg/L, Cr = 0.026 mg/L, As = 0.0006 mg/L, and Hg < 0.00009 mg/L. (^c^) Xuefeng Reservoir (Hancheng District, Weinan City, China): Zn < 0.05 mg/L, Zn < 0.05 mg/L, Fe < 0.03 mg/L, Mn < 0.01 mg/L, Pb < 0.0025 mg/L, Cd < 0.0005 mg/L, Cr = 0.007 mg/L, As = 0.0003, and Hg = 0.00008 mg/L. (^d^) Hexi (Lingwei District, Weinan City, China): Zn < 0.05 mg/L, Fe < 0.03 mg/L, Mn < 0.01 mg/L, Pb < 0.0025 mg/L, and Cd < 0.0005 mg/L. ** Mean ± standard deviation.

**Table 4:** Program for temperature elevation of graphite furnace.

Step	Temperature (°C)	Ramp Time (s)	Hold Time (s)	Internal Flow (mL/min)
Drying 1	110	1	30	250
Drying 2	130	15	30	250
Ashing	1300	10	20	250
Atomization	2200	0	5	0
Cleaning	2450	1	3	250

## Data Availability

The original contributions presented in this study are included in the article. Further inquiries can be directed to the corresponding author(s).
